# Prognosis and Associated Factors among Elderly Patients with Small Artery Occlusion

**DOI:** 10.1038/s41598-019-51671-2

**Published:** 2019-10-25

**Authors:** Yuguang Zhao, Chunxiao Yang, Xiaobo Yan, Xu Ma, Xiaokun Wang, Chunying Zou, Shuang Wang

**Affiliations:** 1 Department of Cell Biology, Harbin Medical University, Harbin, 150081 China; 2 Department of Neurology, The Second Affiliated Hospital of Harbin Medical University, Harbin, 150001 China; 3 Department of Neurology, The First Affliated Hospital of Jiamusi University, Jiamusi, 154000 China

**Keywords:** Outcomes research, Stroke, Risk factors

## Abstract

Small artery occlusion (SAO) is the one of the primary subtype of ischemic stroke in China. However, its outcomes among elderly patients are unclear. Consecutive patients with SAO were recruited at Jiamusi University First Hospital, China between January 2008 and December 2016. Stroke subtype, severity, and risk factors were collected; outcomes at 3, 12, and 36 months after stroke onset were assessed. A total of 1464 SAO patients were included in this study. Participants aged ≥75 years had higher dependency rates than Participants aged <75 years with SAO in all three follow-up periods, in addition to a higher recurrence rate at 12 months and a higher mortality rate 36 months after stroke. After adjusting for confounders, elevated triglyceride level was found to be a protective factor against mortality 36 months after stroke. Stroke severity, diabetes mellitus, artery stenosis, gender, obesity, and high-density lipoprotein cholesterol level were independently associated with the risk of dependency; elevated triglyceride level was an independent risk factor for recurrence at 3 months point after stroke onset. These findings suggest that it is vital to manage risk factors that may affect prognosis of stroke among elderly patients with SAO to improve patient prognosis and reduce the burden of stroke in China.

## Introduction

Stroke has been the primary cause of death and serious, long-term disability worldwide^1^. Although there has shown a decline trend in stroke incidence in developed countries over the last 30 years^2,3^, stroke gradually raised to the leading cause of death and disability from 1990 to 2017 in China, accounting for almost one-third of the total number of deaths from stroke worldwide^4–6^.

Small artery occlusion (SAO) is one of the ischemic stroke subtypes categorized by the Trial of ORG10172 in Acute Stroke Treatment (TOAST)^7^. Previous study reported that in developing countries, SAO accounting for 16–22% of ischemic stroke cases, the percentage higher 4% than developed countries;^8^ however, the latest research showed that SAO accounts for 27.3% of ischemic stroke cases in China, higher than the world level^9^. It is crucial to identify the different potential underlying causes of stroke subtype to targeted treatment and decrease the burden of stroke in China.

However, little is known about outcomes and risk factors associated with outcomes among elderly patients with SAO among Chinese elderly patients. Thus, in this study, we aimed to assess the clinical features, outcomes, and relevant risk factors 3, 12, and 36 months after stroke onset among elderly patients with SAO in China.

## Results

### Patient selection

**Figure 1:**
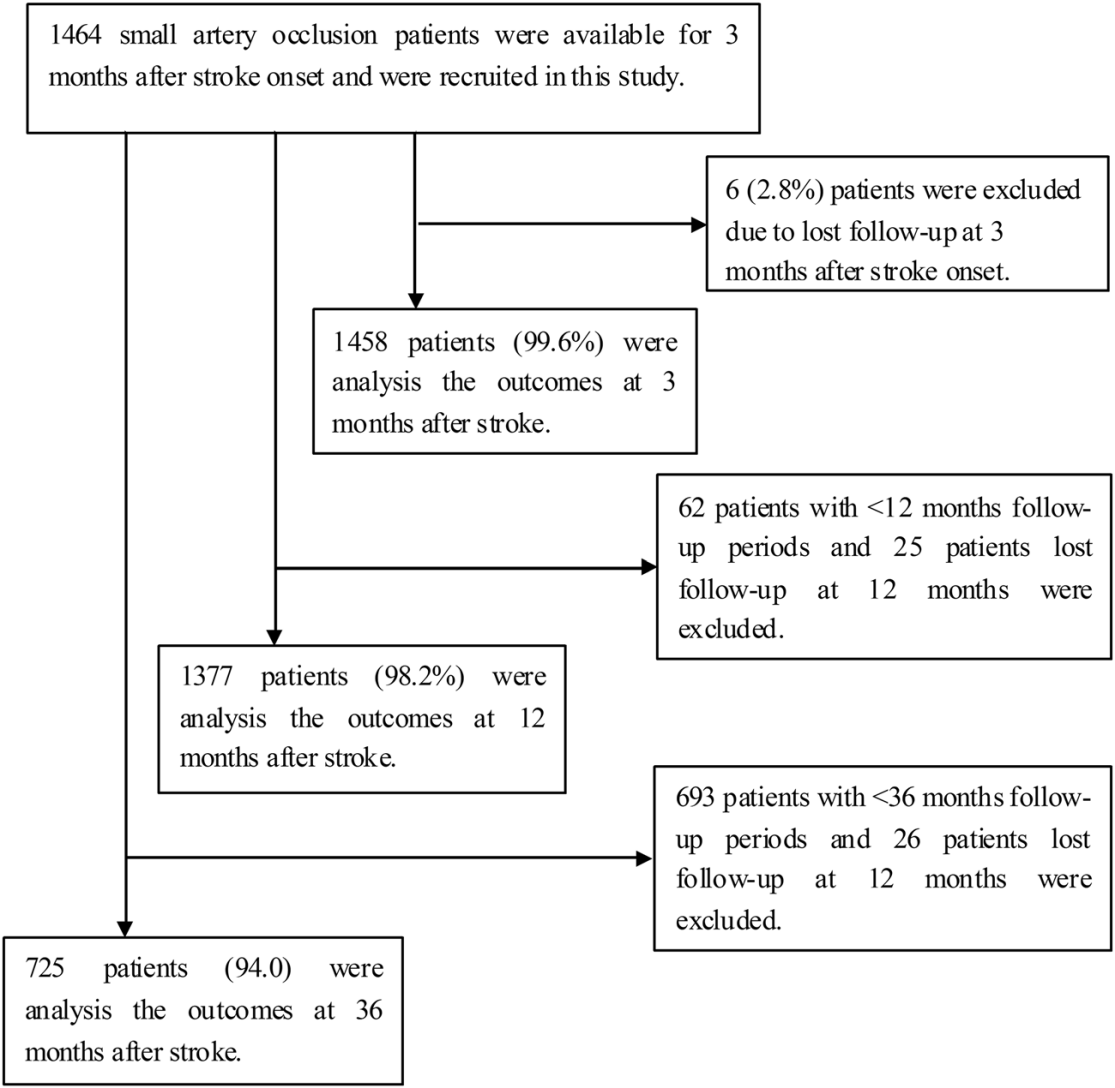
Flow diagram of participants. A total of 1464 patients with SAO were recruited in this study during the study period. Of them, 1458 patients were available for follow up 3 months after stroke onset, resulting in a response rate of 99.6%. Among the 1402 patients who were available >12 months after stroke onset, 1377 patients (98.2%) were followed up at 12 months. Similarly, there were 725 patients (94.0%) available 36 months after stroke onset, after excluding 46 patients who were lost to follow-up during this period.

A total of 1464 patients with SAO (mean age, 63.60 ± 11.43 years) were available for 3 months after stroke onset and were recruited in this study. Of them, 1458 patients were available for follow up 3 months after stroke onset, resulting in a response rate of 99.6%. Among the 1402 patients who were available >12 months after stroke onset, 1377 patients (98.2%) were followed up at 12 months. Similarly, there were 725 patients (94.0%) available 36 months after stroke onset, after excluding 46 patients who were lost to follow-up during this period (Fig. 1).

### Clinical features among SAO patients by age

Of the 1464 SAO patients included in this study at baseline, 1174 (80.2%) patients were aged <75 years and 290 (19.8%) were aged ≥75 years. Compared to aged <75 years group, elderly patients (aged ≥75 years) with SAO were more likely to be female (26.1% vs. 17.1%; P < 0.001), have severe stroke (4.8% vs. 1.4%; P = 0.00019), and have artery stenosis (13.8% vs. 9.5%; P < 0.001) compared to young patients. The prevalence rates of hypertension, atrial fibrillation, obesity, current smoking, and alcohol consumption were significantly higher in the elderly group than in the young group; similar trends were observed with levels of FPG, TG, and LDL-C (Table 1).

**Table 1:** Descriptive characteristics in clinical features and risk factors among patients with SAO by gender groups.

Characteristics	<75 Years	≥75 Years	P
Participants, n (%)	1174 (80.2)	290 (19.8)	
Men	846 (82.9)	174 (17.1)	<0.001
Women	328 (73.9)	116 (26.1)	
Stroke severity:			
Mild	993 (84.6)	219 (75.5)	0.00025
Moderate	165 (14.1)	57 (19.7)	0.017
Severe	16 (1.4)	14 (4.8)	0.00019
Risk factors, n (%):			
Hypertension	910 (77.5)	206 (71.0)	0.020
Diabetes	329 (28.9)	81 (27.9)	0.750
Atrial fibrillation	22 (1.9)	19 (6.6)	<0.001
Artery stenosis	112 (9.5)	40 (13.8)	0.033
Obesity	122 (10.4)	36 (12.4)	0.320
Current smoking	509 (43.4)	54 (18.6)	<0.001
Alcohol drinking	271 (23.1)	16 (5.5)	<0.001
mRS	2.47 (0.11)	2.84 (0.21)	0.099
Laboratory testing:			
FPG, mmol/L, means (SE)	6.14 (0.08)	5.80 (0.12)	0.020
TC, mmol/L, means (SE)	4.57 (0.02)	4.48 (0.04)	0.052
TG, mmol/L, means (SE)	1.45 (0.02)	1.15 (0.03)	<0.001
HDL-C, mmol/L, means (SE)	1.04 (0.01)	1.18 (0.08)	0.076
LDL-C, mmol/L, means (SE)	2.82 (0.02)	2.71 (0.04)	0.007
HCY, mmol/L, means (SE)	15.55 (0.34)	15.13 (0.54)	0.517
CRP, mmol/L, means (SE)	6.52 (0.74)	7.89 (1.00)	0.386

### Differences in outcomes at 3, 12, and 36 months after stroke onset between elderly patients and young patients

Table 2 shows that compared to the young group, there were significantly higher dependency rates at all 3 follow-up periods in the elderly group, with dependency rates of 30.3%, 25.6%, and 46.0% in the elderly group, and 14.9%, 16.9%, and 34.3% in the young group at 3, 12, and 36 months, respectively. Moreover, the recurrence rate was significantly higher in the elderly group than in the young group 12 months after stroke onset (18.3% vs. 13.5%; P = 0.049). Compared to the young group, the elderly group had a worse mortality rates 36 months after stroke, with corresponding rates of 12.6% in the elderly group and 5.4% in the young group.

**Table 2:** Differences in outcomes among patients with SAO during follow-up periods between elderly and young group.

Outcomes	<75 Years	≥75 Years	P
3 months after stroke:			
Mortality	13 (1.1)	4 (1.4)	0.758
Recurrence	51 (4.4)	20 (6.9)	0.074
Dependency	172 (14.9)	86 (30.3)	<0.001
12 months after stroke:			
Mortality	32 (2.9)	13 (4.9)	0.089
Recurrence	149 (13.5)	48 (18.3)	0.049
Dependency	182 (16.9)	64 (25.6)	0.001
36 months after stroke:			
Mortality	32 (5.4)	16 (12.6)	0.003
Recurrence	200 (33.5)	50 (39.4)	0.207
Dependency	194 (34.3)	52 (46.0)	0.018

### Univariate analysis of the association of outcomes with risk factors among elderly patients with SAO

The univariate analysis showed that DM and levels of FPG, TG, HCY, and CRP were associated with mortality at 3, 12, and 36 months. Stroke severity, DM, artery stenosis, obesity, and gender were significantly associated with dependency among elderly SAO patients. Moreover, artery stenosis, HCY level, and gender were associated with recurrence among elderly patients with SAO (Table 3).

**Table 3:** Association of outcomes with risk factors among elderly patients with SAO.

Characteristics	3 Months			12 Months			36 months		
	Mortality	Dependency	Recurrence	Mortality	Dependency	Recurrence	Mortality	Dependency	Recurrence
Gender:									
Men	4 (2.3)	55 (32.4)	12 (6.9)	11 (6.9)	39 (26.2)	27 (16.9)	10 (12.0)	40 (54.8)^*^	38 (45.8)^*^
Women	0	31(27.2)	8 (7.0)	2 (1.9)	25 (24.8)	21 (20.4)	6 (13.6)	12 (30.0)	12 (27.3)
Stroke severity:									
Mild	4 (1.8)	36 (16.9)^*^	12 (5.5)	8 (4.0)	34 (17.8)^*^	32 (16.1)	14 (14.9)	36 (43.9)	38 (40.4)
Moderate	0	36 (63.2)	6 (10.5)	3 (6.0)	20 (42.6)	12 (24.0)	0	14 (48.3)	12 (41.4)
Severe	0	14 (100)	2 (14.3)	2 (14.3)	10 (83.3)	4 (28.6)	2 (50.0)	2 (100)	0
Risk factors:									
Hypertension	2 (1.0)	60 (29.7)	14 (6.9)	11 (5.8)	51 (28.7)	40 (21.2)^*^	12 (12.6)	40 (47.1)	38 (40.0)
No- Hypertension	2 (2.4)	26 (31.7)	6 (7.1)	2 (2.7)	13 (18.1)	8 (10.8)	4 (12.5)	12 (42.9)	12 (37.5)
Diabetes	0	33 (40.7)^*^	8 (9.9)	0^*^	20 (26.7)	13 (17.3)	4 (10.3)	16 (43.2)	16 (41.0)
No-Diabetes	4 (1.9)	53 (26.1)	12 (5.8)	13 (6.9)	44 (25.1)	35 (18.6)	12 (13.6)	36 (47.4)	34 (38.6)
Atrial fibrillation	0	7 (36.8)	0	0	3 (17.6)	3 (17.6)	0	0	0
No-Atrial fibrillation	4 (1.5)	79 (29.8)	20 (7.4)	13 (5.3)	61 (26.2)	45 (18.3)	16 (12.8)	52 (46.8)	50 (40.0)
Artery stenosis	0	24 (60.0)^*^	6 (15.0)^*^	0	15 (42.9)^*^	8 (22.9)	2 (10.5)	8 (47.1)	8 (42.1)
No-Artery stenosis	4 (1.6)	62 (25.4)	14 (5.6)	13 (5.7)	49 (22.8)	40 (17.5)	14 (13.0)	194 (34.3)	42 (38.9)
Obesity	0	21 (58.3)^*^	4 (11.1)	2 (5.6)	8 (23.5)	6 (16.7)	2 (11.1)	12 (75.0)^*^	10 (55.6)
No-Obesity	4 (1.6)	65 (26.2)	16 (6.3)	11 (4.8)	56 (25.9)	42 (18.5)	14 (12.8)	40 (41.2)	40 (36.7)
Current smoking	0	19 (35.2)	6 (11.1)	4 (7.4)	13 (26.0)	12 (22.2)	4 (14.3)	14 (58.3)	16 (57.1)^*^
No-Current smoking	4 (1.7)	67 (29.1)	14 (6.0)	9 (4.3)	51 (25.5)	36 (17.2)	12 (12.1)	38 (42.7)	34 (34.3)
Alcohol drinking	0	6 (37.5)	2 (12.5)	2 (12.5)	0^*^	4 (25.0)	2 (20.0)	4 (50.0)	6 (60.0)
No-Alcohol drinking	4 (1.5)	80 (29.9)	18 (6.6)	11 (4.5)	64 (27.1)	44 (17.8)	14 (12.0)	48 (45.7)	44 (37.6)
FPG, mmol/L									
Yes	13.53 (0)^*^	5.98 (0.24)	6.16 (0.59)	7.86 (1.34)	5.95 (0.32)	6.21 (0.37)	6.65 (1.15)	5.96 (0.32)	5.74 (0.30)
No	5.73 (0.11)	5.63 (0.13)	5.78 (0.13)	5.71 (0.12)	5.63 (0.12)	5.70 (0.14)	5.89 (0.21)	5.84 (0.27)	6.12 (0.31)
TC, mmol/L									
Yes	3.82 (0.27)	4.58 (0.09)	4.65 (0.21)	4.18 (0.20)	4.48 (0.11)	4.54 (0.14)	4.22 (0.20)	4.33 (0.12)	4.33 (0.12)
No	4.49 (0.04)	4.45 (0.05)	4.46 (0.04)	4.49 (0.05)	4.49 (0.05)	4.46 (0.05)	4.41 (0.07)	4.48 (0.09)	4.42 (0.08)
TG, mmol/L									
Yes	0.82 (0.13)	1.17 (0.06)	1.43 (0.15)	0.80 (0.08)^*^	1.22 (0.08)	1.25 (0.09)	0.88 (0.50)^*^	1.16 (0.08)	1.22 (0.07)
No	1.16 (0.03)	1.15 (0.04)	1.13 (0.03)	1.15 (0.08)	1.13 (0.03)	1.11 (0.03)	1.19 (0.05)	1.22 (0.06)	1.11 (0.06)
HDL-C, mmol/L									
Yes	1.04 (0.04)	1.40 (0.25)	1.03 (0.10)	1.09 (0.06)	1.01 (0.04)	1.03 (0.05)	2.96 (1.28)	1.04 (0.03)	1.02 (0.03)
No	1.18 (0.08)	1.08 (0.02)	1.19 (0.08)	1.19 (0.09)	1.26 (0.12)	1.22 (0.10)	1.10 (0.02)	1.15 (0.03)	1.53 (0.27)
LDL-C, mmol/L									
Yes	2.31 (0.20)	2.80 (0.07)	2.82 (0.12)	2.56 (0.19)	2.79 (0.09)	2.79 (0.10)	2.40 (0.14)	2.63 (0.09)	2.63 (0.09)
No	2.72 (0.04)	2.69 (0.04)	2.71 (0.04)	2.73 (0.04)	2.71 (0.04)	2.70 (0.04)	2.64 (0.06)	2.65 (0.08)	2.59 (0.07)
HCY, mmol/L									
Yes	12.25 (0.66)	16.38 (1.23)	22.98 (4.32)	12.01 (0.62)^*^	15.23 (1.54)	16.24 (1.99)	13.37 (0.84)^*^	16.96 (1.30)	15.82 (1.59)
No	15.16 (0.55)	14.63 (0.58)	14.53 (0.48)	15.49 (0.62)	15.58 (0.65)	15.11 (0.57)	16.18 (1.05)	15.29 (1.34)	15.83 (1.13)
CRP, mmol/L									
Yes	37.63 (20.71)	8.40 (1.21)	7.71 (1.56)	18.35 (7.00)	5.92 (0.78)	7.74 (0.98)	30.19 (8.77)^*^	8.45 (1.68)	11.66 (2.81)
No	7.47 (0.96)	7.07 (1.27)	7.94 (1.07)	7.66 (1.05)	8.22 (1.37)	8.31 (1.28)	6.56 (0.89)	7.75 (2.12)	8.16 (1.67)

Data were presented by n (%) for categorized variates, by means (standard error) for continued variates. *indicated P < 0.05 compared between two groups.

### Multivariate analysis of the association of outcomes with risk factors among elderly patients with SAO

After adjusting for confounders, including gender, stroke severity, risk factors, and laboratory measurements, the relationship between risk factors and mortality at 3 and 12 months leveled out, but TG level was a protective factor against mortality 36 months after stroke. The risk of long-term mortality among elderly patients with SAO decreased by 95% for every 1 mmol/L increase in TG levels (P = 0.023). Stroke severity, DM, artery stenosis, gender, obesity, and HDL-C level were independently associated with the risk of dependency. Of these factors, stroke severity, DM, and artery stenosis were determinants of dependency at 3 months; stroke severity and artery stenosis at 12 months; and gender, obesity, and level of HDL-C at 36 months. Only high TG level was an independent risk factor for recurrence 3 months after stroke onset, with an RR (95% CI) of 2.32 (1.10, 4.89; P = 0.027; Table 4).

**Table 4:** Determinants of outcomes among elderly patient s with SAO. (* indicated P < 0.05).

Characteristics	Reference	3 Months			12 Months			36 months		
		Mortality	Dependency	Recurrence	Mortality	Dependency	Recurrence	Mortality	Dependency	Recurrence
Women	Men	—	—	—	—	—	—	—	4.23 (1.63,10.98)*	1.94 (0.86,4.39)
Stroke severity:	Mild	—		—	—		—		—	—
Moderate		—	11.74 (5.67,24.30)*	—	—	4.14 (2.01,8.53)*	—	0	—	—
Severe		—	0	—	—	18.77 (3.86,91.19)*	—	0.99 (0.10,9.36)	—	—
Hypertension	No	—	—	—	—	—	2.22(0.98,4.99)	—	—	—
Diabetes	No	—	2.20 (1.07,4.50)*	—	0	—	—	—	—	—
Artery stenosis	No	—	5.64 (2.42,13.14)*	2.65 (0.94,7.49)	—	2.34 (1.02,5.37)*	—	—	—	—
Obesity	No	—	2.32 (0.96,5.61)	—	—	—	—	—	8.63 (2.19,34.01)*	—
Smoking	No	—	—	—	—	—	—	—	—	2.18 (0.91,5.26)
Drinking	No	—	—	—	—	0	—	—	—	—
TG	—	—	—	2.32 (1.10,4.89)*	0.12 (0.01,0.95)	—	—	0.05 (0.00,0.57)*	—	—
HDL-C	—	—	—	—	—	—	—	—	0.07 (0.01,0.43)*	—
HCY	—	—	—	—	0.90 (0.79,1.03)	—	—	0.92 (0.82,1.04)	—	—
CRP	—	—	—	—	—	—	—	1.05 (1.02,1.09)*	—	—

## Discussion

To our knowledge, this is the first study to assess the clinical characteristics, outcomes, and risk factors associated with outcomes among elderly patients with SAO 3, 12, and 36 months after stroke onset in China. Compared to young patients, elderly patients with SAO were more likely to be female, have severe stroke, and have artery stenosis. The elderly group was found to have a poor prognosis; the long-term mortality and dependency rates at all 3 follow-up periods, and the recurrence rate at 12 months were significantly higher in the elderly group compared to the young group. Moreover, TG level was a protective factor against long-term mortality; stroke severity, DM, artery stenosis, gender, obesity, and HDL-C level were independently associated with the risk of dependency. In addition, TG level was an independent risk factor for recurrence 3 months after stroke onset.

Previous studies have demonstrated that elderly people have a worse prognosis after stroke^10,11^. In this study, we also observed that elderly group more prone to poor prognosis; the long-term dependency rates in the aged ≥75 years group at all 3 follow-up periods were significantly higher than aged <75 years group (30.3% vs 14.9%; 25.6% vs 16.9%; 46.0% vs 34.3%, respectively).

Several studies showed that low-level of cholesterol was significantly associated to the poor outcome after acute ischemic stroke^12–14^. Similarly, a previous study showed that a lower cholesterol level was associated with an increased risk of death among elderly patients with ischemic stroke^15^. Patients with hyper-cholesterolemia, especially older patients, had poor recovery whether in short-term or in long-term after stroke^16^.

The relationship between TG level and outcomes after stroke is unclear. In this study, high TG level was a protective factor against long-term mortality, but was an independent risk factor for recurrence 3 months after stroke onset. Stroke severity, DM, artery stenosis, gender, obesity, and HDL-C level were independently associated with the risk of dependency. The opposing relationships between TG level and long-term mortality and between TG level and short-term recurrence among elderly patients with SAO may be explained by medication use. Statin use may improve mortality rates 36 months after stroke, but not recurrence rates 3 months after stroke.

The inflammatory response plays an important role in the occurrence and development of cerebral ischemia^17^. CRP is a marker of inflammation associated with plaque instability, stenosis, or occlusion of the small vessels^18,19^. The association of CRP level with stroke outcome is currently unclear. One study found that a high level of CRP was a significant predictor of prognosis after lacunar stroke^20^. Furthermore, elevated CRP may increase the risk of poor prognosis in patients with SAO aged <75 years^21^. In line with these findings, we found that an elevated level of hs-CRP was associated with increased long-term mortality among elderly patients with SAO.

Previous study has demonstrated that age and stroke severity were associated with prognoses (mortality and recurrence) after stroke^22^. DM was also associated with higher mortality, dependency, and recurrence rates after stroke^23,24^. Consistent with these studies, stroke severity, DM, artery stenosis, gender, obesity, and HDL-C level were independently associated with the risk of dependency in the present study.

There were several limitations to this study. First, this study was conducted in a single hospital, and thus may not represent the general patient population. Second, data regarding the control of risk factors following stroke were lacking from this study, and this may have affected the evaluation of predictors of outcomes. Third, those patients followed-up by telephone did not reported separately. This may impact the results. Finally, patients with mild stroke may have been excluded from this study if they did not present at the hospital, and this may have affected the analysis.

In conclusion, this is the first study to assess the clinical characteristics, outcomes, and relevant factors associated with outcomes among elderly patients with SAO at 3, 12, and 36 months after stroke onset. The long-term mortality and dependency rates in all 3 follow-up periods, and the recurrence rate at 12 months were significantly higher in the elderly group compared to the young group. Stroke severity, DM, artery stenosis, gender, obesity, and HDL-C level were independently associated with the risk of dependency. Moreover, Elevated TG level was a protective factor against long-term mortality and an independent risk factor for recurrence 3 months after stroke onset. These findings suggest that it is crucial to manage risk factors associated with stroke outcome among elderly patients with SAO to improve patient prognosis and reduce the burden of stroke in China.

## Methods

### Patient selection

All patients in this study were identified from the stroke registry of the Department of Neurology, Jiamusi University First Hospital, China between January 2008 and December 2016. The inclusion criteria of participants and study design have been described previously^12^. In brief, all consecutive patients with first-ever acute ischemic stroke who were admitted to the Department of Neurology, Jiamusi University First Hospital within 72 hours of stroke onset were recruited for this study. Detailed information, including clinical features and stroke risk factors, were collected.

All investigative protocols were approved by the ethics committee of Jiamusi University First Hospital; the methods were carried out in accordance with the approved guidelines, and informed consent was obtained from each participant or their next-of-kin.

### Data collection

Data collection and outcome evaluation were performed by senior neurologists who used standardized variable definitions and scores. According to TOAST classification, cases were categorized into large artery atherothrombotic, cardioembolic, SAO, other causes, and undetermined on admission^7^. Stroke severity was grouped into three groups according to National Institutes of Health Stroke Scale (NIHSS) scores: mild (NIHSS score: ≤7), moderate (NIHSS score: 8–16), and severe (NIHSS score:≥17)^25^.

Conventional risk factors, including hypertension, diabetes mellitus (DM), atrial fibrillation, intracranial artery stenosis, smoking, and alcohol consumption, were defined according to self-reported previous medical history or examination on admission; obesity was defined as a body mass index ≥28 kg/m^2^ ^26^. The NIHSS score was evaluated on admission and at discharge; the modified Rankin Scale (mRS) score was assessed on admission, at discharge, and at 3, 12, and 36 months after stroke.

### SAO definition and outcomes assessment

Stroke events were defined according to the World Health Organization’s criteria, and all cases of stroke were confirmed using neuroimaging^27^. All acute ischemic stroke patients diagnosed with SAO according to TOAST classification were included in this study.

Outcomes included mortality, dependency, and recurrence rates at 3, 12, and 36 months after stroke. Mortality was defined as all-cause cumulative death at the corresponding follow-up time point. Recurrence was defined as all new-onset vascular events, including stroke, myocardial infarction, and venous thrombosis. Dependency was defined as an mRS score >2^28^.

### Follow-up

Follow-up was conducted by the same senior neurologist at 3, 12, and 36 months after stroke onset. All patients were followed up as outpatients through face-to-face interviews, except for patients followed up by telephone. The mRS score was assessed for each patient during the follow-up period.

### Statistical analysis

All SAO patients were divided into two groups: age <75 years and ≥75 years. Continuous variables, including age, NIHSS score, Barthel Index, mRS score, and laboratory measurements of fasting plasma glucose (FPG), total cholesterol, triglycerides (TG), high-density lipoprotein cholesterol (HDL-C), low-density lipoprotein cholesterol (LDL-C), HCY, and CRP levels, are presented as means with standard deviations (or as medians with ranges where appropriate), and were compared between groups using the Student’s t-test or the Mann-Whitney U test. Categorized variables, including stroke severity, previous medical history, risk factors, and outcomes, are presented as rates or frequencies, and were compared between groups using the chi-squared test. The determinants of outcomes were analyzed using logistic regression after adjusting for the confounders of stroke severity, previous medical history, and risk factors; results are presented using adjusted relative risk (RR) with 95% confidence intervals (CIs). All statistical analyses were performed using SPSS version 19.0 (SPSS Inc., Chicago, IL), and a two-tailed P value < 0.05 indicated statistical significance.
